# Neuroplacentology of stress: Novel frontiers linking maternal mental health to offspring neurodevelopment

**DOI:** 10.1016/j.ynstr.2025.100778

**Published:** 2025-12-11

**Authors:** Cristiana Cruceanu

**Affiliations:** Department of Physiology and Pharmacology, Karolinska Institutet, Stockholm, Sweden

**Keywords:** Placenta-brain, Perinatal stress, Neuropharmacology, Neurodevelopment

## Abstract

Neuroplacentology is an emerging interdisciplinary field integrating molecular neuroscience, placental biology, environmental modeling, and single-cell techniques to study stress-related neurodevelopmental programming. The placenta, once considered merely a conduit between mother and fetus, is now recognized as an important regulator of fetal brain development and a mediator of prenatal maternal stress and other exposures. To highlight this important aspect of the neurobiology of stress, this review outlines how maternal stress, genetic susceptibility, and environmental exposures converge at the placenta-brain axis to influence offspring psychiatric vulnerability.

Risk and resilience for psychiatric conditions are shaped by interactions between genetic predisposition and environmental exposures, including during the highly plastic prenatal period. Prenatal stress exposure can alter neuronal differentiation, transcription factor gene-regulatory networks, and excitation/inhibition neuronal balance. In parallel, maternal metabolic disorders, placental endocrine dysregulation, and psychotropic medication exposure modulate neuroactive pathways critical to brain development. The placenta responds to these exposures and synthesizes key stress molecules such as corticotropin-releasing hormone, serotonin and other neuromodulators, highlighting its neuroregulatory role.

To exemplify the promising future of neuroplacentology for our understanding of perinatal health and stress research, this review highlights innovative methodological approaches such as human-specific placental organoid systems and single-cell multi-omics. I propose that future research should focus on identifying placental biomarkers predictive of neurodevelopmental outcomes and refining *in-vitro* models for testing pharmacological interventions in a non-invasive manner. Elucidating the mechanisms at the placenta-brain interface, could lead to a better understanding of the developmental origins of mental illness and inform early intervention strategies.

## Graphical abstract

**Figure:**
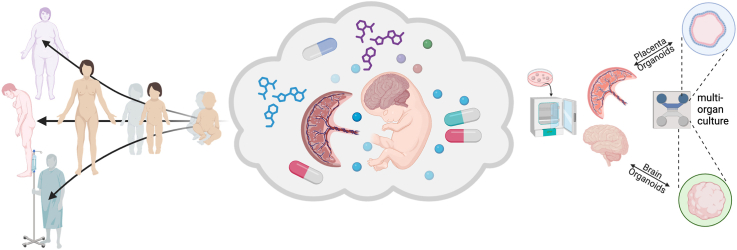

## 1 Introduction

Neurodevelopmental and psychiatric phenotypes such as autism spectrum disorders, schizophrenia and even stress-related disorders like depression and anxiety often have causal roots early in development, where genetic and environmental factors intersect. Stress-related psychiatric disorders remain among the leading causes of global disease burden. While some level of genetic predisposition has been established, the complexity of these disorders lies in their multifactorial nature, whereby environmental factors, particularly early-life stress exposure, exert long-lasting effects on brain development and function, subsequently contributing to pathology. Recent interdisciplinary advances have enabled a mechanistic understanding of how stress, especially related to glucocorticoid signaling, interacts with genetic susceptibility to shape psychiatric trajectories. In this perspective review I will synthesize my contributions within the seminal body of work in this field, emphasizing the integrative role of genetic variation, prenatal stress exposure, and neurodevelopmental programming. I will especially focus on my view on the imperative need to understand the symbiosis between two key fetal tissues, the placenta and brain, which I believe could help fill the gaps in our understanding of the developmental origins of disease and be harnessed for treatment and prevention.

The placenta, historically overlooked in this context, is now emerging as a key interface between maternal and fetal biology, especially under conditions of prenatal stress (Monk et al., 2016; Monk et al., 2019). In this perspective I aim to explore the placenta-brain axis within the emerging field of neuroplacentology (Kratimenos and Penn, 2019), underscoring its relevance to stress neurobiology and advocating for a research paradigm that bridges neuro-development and neuro-endocrinology. The advent of advanced human-specific models such as trophoblast and neural organoids, combined with single-cell ‘omics approaches, positions us to achieve future discoveries in stress neurobiology that were not previously possible using non-human animal models or in overly simplified human cell systems. I will make the case for the value of these novel human-specific *in-vitro* tools in pushing the boundaries of our understanding as well as leading us closer to the possibility of personalized medicine in psychiatry.

## 2 Current considerations and state-of-the-art

### 2.1 Mental illness and stress during the perinatal period

Long-term stress over-exposure as in the case of mental illness, can turn an adaptive physiological response like the stress response maladaptive. This is particularly important in pregnancy, when chronic stress poses a health risk for both mother and developing baby. Prenatal stress exposure in situations of maternal mental illness is among the most significant early-life risk factors for offspring psychiatric disorders (Stein et al., 2014; Van den Bergh et al., 2020). Epidemiological data shows that the most prevalent prenatal maternal mental illnesses are depression and anxiety disorders, which affect 25 % or more women worldwide (Field, 2017; Caffieri et al., 2024, Aziz et al., 2025). Women of reproductive age are particularly vulnerable to these psychopathologies and importantly, pregnancy can exacerbate the clinical presentation in women with history of mental illness, or pose an increased risk level for developing a first episode of depression or anxiety in over 30 % of pregnant women (Yan et al., 2020). In addition to clinical diagnoses, nearly 75 % of postpartum mothers reported at least one major stressful event in the year leading up to delivery (Burns et al., 2015). Psychosocial, cultural and environmental stressors experienced during gestationcan be detrimental to the progression of pregnancy as well as the health of the mother and child (Cruceanu et al., 2017; Krontira et al., 2020). The notion of ‘prenatal stress’ as a research construct is conceptualized in several ways in the human literature, reflecting the diversity of stressors possibly experienced during gestation. Examples of psychosocial stressors include changes in personal life, job status, housing, domestic violence and family structure (Orr et al., 1996). The more general term ‘psychosocial stress’ refers to stressful things that happen whether a person is pregnant or not (daily hassles, financial or marital strain, social stress). Conversely, pregnancy-specific distress and anxiety refer to anxiety about things that are directly connected to the pregnancy, such as worries about the outcome of prenatal screenings, fears about infant health and development, and general uncertainly about how motherhood will change one's life (Lobel et al., 2008). Strong evidence supports that both components of prenatal stress – psychosocial stress and pregnancy-specific distress – can have marked effects on pregnancy and offspring development (Lupien et al., 2009; Van den Bergh et al., 2020). Therefore, it is important to understand the biological underpinnings of these early-life stressors, and how they can contribute to causing pathology, as well as give clues for future interventions.

### 2.2 Genetic susceptibility to psychiatric disorders

When considering susceptibility or resilience to stress, genetic factors should be considered. The genetic landscape of mood disorders is marked by both common variants of small effect and rare, high-impact mutations. Pivotal contributions to this domain in mental illness have been made in the last decade, with advances in genotyping and sequencing technologies leading to a genomics revolution (Brainstorm et al., 2018; Cruceanu et al., 2018, Coleman et al., 2020). This underscored that psychiatric disorders involve complex polygenic risk architecture (Musliner et al., 2019; Amare et al., 2023). Psychiatric disorders in general, including schizophrenia (SCZ), bipolar disorder (BD), major depressive disorder (MDD), autism spectrum disorder (ASD), and attention deficit hyperactivity disorder (ADHD) have been shown through twin studies to be highly heritable, with genetic variance explaining from 34 % to 77 % of phenotypic variance (Polderman et al., 2015; Bourque et al., 2024). Given that none of these disorders are 100 % heritable, an important consideration should be made for the role played by the environment alone or in interaction with the genetic landscape. Despite knowing the heritability estimates and many of the risk loci, an enduring challenge is understanding the molecular mechanisms of how genetic susceptibilities interact with prenatal stress or other environmental exposures. Interestingly, many loci identified as associated with mental illness susceptibility reside in non-coding regions (Lopez et al., 2014; Cruceanu et al., 2015; Lopez et al., 2015) with likely gene-regulatory roles such as enhancers, promoters and miRNAs. This hints at the potential modulatory role of environmental factors acting on these loci rather than only dysfunctional proteins that would be encoded through coding variants in the DNA. Post-mortem investigations have deepened this genetic perspective by linking gene expression patterns to psychiatric outcomes. For instance, transcriptomic studies of post-mortem brain samples from donors who lived with mental illness and passed from suicide have identified consistent downregulation of stress-adaptive genes, particularly within the prefrontal cortex and amygdala (Lopez et al., 2014; Cruceanu et al., 2015).

### 2.3 Environmental programming via glucocorticoid exposure in neurodevelopment

Genetic variations often co-occur with epigenetic modifications such as DNA methylation (Huzayyin et al., 2014; Cruceanu et al., 2016) and histone modification changes (Cruceanu et al., 2013), suggesting a complex and inter-connected molecular signature of stress-exposure. The hypothalamic-pituitary-adrenal (HPA) axis is a central component of the body's neuroendocrine response to environmental stress. Glucocorticoids, the end-product of HPA activation, cross placental barriers and shape brain development during critical prenatal periods in both adaptive and maladaptive ways (Lupien et al., 2009). In recent studies, we have demonstrated that prolonged glucocorticoid receptor (GR) activation biases brain developmental pathways toward an overproduction of inhibitory or excitatory neurons, with critical periods of exposure closely aligning with the orchestrated trajectory of brain development (Cruceanu et al., 2022; Krontira et al., 2024; Dony et al., 2025). This occurred through GR-dependent transcriptional networks, with implications for altered excitation/inhibition balance – a vulnerability mechanism in neurodevelopmental psychopathologies like ASD and schizophrenia (Bruining et al., 2020; Satterstrom et al., 2020). Moreover, acute glucocorticoid exposure during critical neurodevelopmental windows triggered pronounced shifts in regional identity and transcriptomic profiles of neurons, reinforcing the idea that transient exposure events may have enduring molecular consequences (Cruceanu et al., 2022). This is consistent with multiple lines of evidence supporting that elevated stress exposure affects HPA-axis maturation and neuronal differentiation (Lupien et al., 2009; Monk et al., 2019; Krontira et al., 2020). Furthermore, prospective cohort studies have demonstrated that maternal stress correlates with altered brain structure and function in offspring, particularly in regions implicated in emotional regulation and stress responsivity (Graham et al., 2018; Graham et al., 2019). These findings underscore the need for mechanistic studies that elucidate how maternal stress signals are transmitted and modulated across the placenta, to reach the developing brain and exert their adaptive and maladaptive effects.

### 2.4 Gene × environment interactions

Emerging from the convergence of genetic and environmental paradigms is the gene-by-environment (G × E) framework, which posits that genetic risk is modulated by environmental exposure to determine phenotypic outcomes. Expression studies in human perinatal tissues and *in-vitro* model systems have revealed stress-induced transcriptional shifts that are modulated by underlying genetic variability (Czamara et al., 2022; Krontira et al., 2024). For instance, certain isoforms or polymorphisms in GR-regulated genes correspond with heightened transcriptional sensitivity to glucocorticoids in placental tissue, offering a developmental explanation for individual differences in vulnerability to psychiatric illness later in life (Clifton et al., 2017). Transcriptomic and epigenomic data from fetal and postnatal tissues have been instrumental in identifying shared molecular signatures of stress exposure through regulatory hubs that control neurodevelopmental programming and stress reactivity (Lopez et al., 2017; Cruceanu et al., 2022; Gerstner et al., 2022; Krontira et al., 2024; Dony et al., 2025). Some prominent examples relate to altered methylation and expression of *NR3C1*, *FKBP5*, *BDNF*, and *SLC6A4* to name a few key genes (Provencal and Binder, 2015; Turecki and Meaney, 2016, Cruceanu et al., 2017).

### 2.5 Perinatal mental illness and metabolic/endocrine health in early life

Beyond epigenomic and transcriptomic regulation, emerging evidence suggests a profound interconnection between metabolic and mental health, particularly during the perinatal period when early-life exposures shape long-term neurodevelopmental trajectories. The placenta-brain axis plays a critical role in mediating maternal metabolic signals to the fetus, influencing neurodevelopmental outcomes through hormonal, inflammatory, and epigenetic mechanisms (Monk et al., 2019). Maternal obesity, gestational diabetes, and dysregulated placental metabolism increase fetal exposure to pro-inflammatory cytokines, hyperglycemia, and altered insulin signaling, which in turn predispose offspring to psychiatric disorders such as ASD, schizophrenia, and depression (Bronson and Bale, 2016). For example, maternal hyperglycemia can induce oxidative stress and mitochondrial dysfunction in the developing brain, leading to disrupted synaptogenesis and altered neurotransmitter systems (Burns et al., 2015, Burton and Fowden, 2015; Bronson and Bale, 2016, Monk et al., 2019). Additionally, metabolic disorders influence fetal HPA axis programming, increasing susceptibility to stress-related psychiatric disorders.

Corticosteroid hormones like cortisol can be chronically dysregulated in response to psychological stress, while also being essential for normal body functions and fetal development. When pregnant women experience severe stress, elevated corticosteroids can affect placenta function, and cross the placenta barrier, leading to fetal overexposure (Krontira et al., 2020). The placenta, a gate-keeper tissue connecting mother and fetus, brings in oxygen and nutrients, and mediates passage of potentially harmful agents from the environment. Some molecules pass freely. Cortisol plays a key role in regulating the temporal progression of organ development events. Perhaps for this reason, its passage across the placenta is tightly controlled through a mechanism involving the 11β-Hydroxysteroid Dehydrogenase Type 2 (11β-HSD2) placental-specific enzyme (Shearer et al., 2019) that converts maternal cortisol to inactive cortisone, effectively reducing passage to the fetal compartment (Stirrat et al., 2018). When these processes are disrupted, aberrant hormone levels can lead to pregnancy disorders and improper fetal maturation. For example, cortisol dysregulation is associated with impaired implantation and function of trophoblast stem cells. In early pregnancy, elevated cortisol has been shown to repress inflammatory genes and impair the formation of chorionic villi, the functional unit of the placenta (Shearer et al., 2019).

Indeed, the placenta is a neuroendocrine organ that orchestrates fetal brain development in early pregnancy. Acting as both a sensor and a regulator, the placenta produces and modulates key hormones that shape neurodevelopmental trajectories, ensuring that the fetal brain receives the biochemical signals necessary for growth, differentiation, and maturation (Burton and Fowden, 2015). This intricate communication network determines the early programming of neural circuits and influences cognitive and behavioral outcomes later in life (Bronson and Bale, 2016). However, disruption of this endocrine balance can have profound consequences, potentially leading to ASD, schizophrenia, and ADHD (Bronson and Bale, 2016). Some of the key placental hormones with neurodevelopmental roles are cortisol, corticotropin-releasing hormone (CRH), and estrogens (Estradiol, Estriol). Importantly, hormones that are essential to placental function such as human chorionic gonadotropin (hCG), progesterone, insulin-like growth factors (IGFs) have also been associated to brain development through neuronal differentiation, proliferation, and myelination. Placental endocrine function can be disrupted leading to abnormal gene expression, infection, or prematurity, resulting in long-term damage from loss of the normal hormonal milieu. Elevated maternal glucose levels can alter placental IGF signaling in gestational diabetes, leading to abnormal brain overgrowth (macrocephaly) and increased autism risk (Dyer et al., 2016).

### 2.6 Neurotransmission and pharmacology during fetal development

Of equal importance to stress pathology in the context of perinatal mental health are psychotropic medications needed for the mother's treatment, of which the most prescribed during pregnancy are the antidepressant class selective serotonin reuptake inhibitors (SSRIs) (Chisolm and Payne, 2016). A large body of epidemiological evidence from Nordic register-based studies has indicated that use of antidepressants is safe in pregnancy regarding lasting child outcomes (Stephansson et al., 2013; Sujan et al., 2017; Rees et al., 2019). Conversely, if antenatal depression is left untreated, the harm to expectant mothers is apparent, leading in the most severe cases to suicide. In fact, women with antenatal depression in Europe have more than 4 times higher risk of suicidal behavior within one year after the diagnosis (Hagatulah et al., 2024). Moreover, maternal antenatal depression has been associated with complications in the offspring such as preterm birth, excessive infant crying, and child mental health problems (Smith et al., 2020). While the need for treatment in the patient is clear, the specific choice of treatment is less so. Even though lasting child outcomes are not well supported, there is a mechanism for these pharmacological agents to reach the fetal brain given that as many as 16 % of exposed babies suffer from some degree of withdrawal symptoms at birth (McLean et al., 2019; Shea et al., 2021). The lasting implications of this early side effect are unclear, and in fact many studies refer to the short duration and self-limiting nature of the serotonin syndrome in newborns, requiring only observation and supportive management during the first 24 h following birth (Rampono et al., 2009; Grigoriadis et al., 2013; Galbally et al., 2017). Therefore, over-interpretation of this outcome should be approached with care given the acute risk of suicide for expectant mothers who do not adhere to recommended treatment. Importantly, studies moving forward should prioritize disentangling the biological effect of maternal stress and pathology – potentially untreated – from the pharmacological interventions that could save her life and consequently lead to better parenting behavior, a well-established child protective factor. With increasing rates of perinatal mental illness, so are there increasingly more pregnant women worldwide being prescribed SSRIs (Molenaar et al., 2020; Robiyanto et al., 2023). Given the underlying stress neurobiology dysregulation, are the effects of added pharmacology likely to be increased risk, or create resilience? Future studies could explore risk-related biomarkers and mechanisms linking the mother and offspring (for example low-grade inflammation), more complex diagnostic criteria and sub-phenotypes of maternal depression severity, and importantly biological mechanisms in a systemic and tissue-specific manner.

Antidepressants directly and indirectly influence monoamine neurotransmitters – serotonin, norepinephrine, and dopamine. Neurotransmission dysregulation in early development could lead to long-term consequences in behavioral and cognitive outcomes (St-Pierre et al., 2016). Among the early factors that regulate the development of brain function through assembly of cortical circuits, the neurotransmitter serotonin is a signal that impacts a broad diversity of cellular processes (Lesch and Waider, 2012). Serotonin is central to the neurobiology of depression and SSRIs function by regulating serotonin levels. Beneficial in pathological states, this modulation may be detrimental when exposure occurs without clinical indication. During development, serotonin is secreted, and a variety of its receptors are dynamically expressed in the embryonic developing cortex in a region- and cell type-specific manner (Lesch and Waider, 2012, Garcia et al., 2019). Building cortical circuits relies on a series of precisely timed events that occur during embryonic and early postnatal development (Lesch and Waider, 2012, Garcia et al., 2019). Critical components include neuronal proliferation, migration, and differentiation.

The placenta plays a central role the temporal regulation of serotonin supply to the developing brain. Disruptions in neurotransmission during critical developmental windows, such as those induced by SSRIs, will significantly alter placental function in addition to fetal brain development, increasing risk for neurodevelopmental disorders. Emerging research supports the concept of a placenta-brain axis, wherein the placenta regulates not only nutrient and oxygen delivery but also neuroactive signaling molecules. Monoamine neurotransmitters serotonin, norepinephrine, and dopamine are produced or modulated by the placenta (Rosenfeld, 2021), and are proposed to play important roles in neurodevelopmental outcomes. In the placenta, monoamines regulate hormone synthesis and affect placental metabolism (Shi and Zhuang, 1993, Elmetwally et al., 2018). Analogously to the brain, the placenta has transport mechanisms that actively take up monoamines into trophoblast cells, the key placental cell types. These transporters are considered to play important roles in the differentiated syncytiotrophoblast layer, but their status and activities in the undifferentiated, progenitor cytotrophoblast cells are as yet unknown (Rosenfeld, 2021). In fact, precise mechanisms of placental neurotransmission and its influence on fetal brain development remain largely unexplored, though it has been proposed that the placenta both synthesizes and secretes these neurotransmitters for transport to the fetus, acting as a supplier for immature early developing brain. This evidence suggests that proper monoamine levels in the feto-placental unit are required to ensure precise fetal development and programming.

### 2.7 Importance of studying human-specific mechanisms in human-specific systems

Environmental exposures are essential to the healthy course of human development, however some of the processes involved are not replicated in commonly used non-primate species like mice and rats (Cruceanu et al., 2017). This applies at genetic, epigenetic, cellular, and molecular regulatory levels. While rodents constitute invaluable models for behavioral and neurodevelopmental phenotypes, some cellular and molecular processes are uniquely human. During brain development, important differences exist between species in the abundance, lineage complexity, and proliferative potential of neural progenitor cells such as the gyrified species-specific basal radial glia, key neural progenitors that are responsible for in ventricle structure, cortical folding, and ultimately brain architecture (Pollen et al., 2015). Additionally, regulatory DNA features such as glucocorticoid response elements and enhancers are not well conserved between humans and rodents (Yue et al., 2014). The latter limits the value of studying transcriptional response to exposures in non-human species. Therefore, it is sometimes essential to use model systems that accurately replicate human cellular and molecular responses. Similarly, the human placenta has unique features from rodents for example in the level of cellular organization, endocrine regulation, and timeline of development across gestation (Soncin et al., 2018).

Advancements in trophoblast (placenta) (Turco et al., 2018; Karvas et al., 2022) and neural (brain) (Birey et al., 2017; Lancaster, 2021) organoids and organotypic (Hendriks et al., 2024) cultures now enable us to model human-specific developmental processes. These *in-vitro* systems provide an unprecedented opportunity to study human physiology and pharmacology in a controlled environment, making them compelling tools for deciphering the impact the environment on the placenta-brain axis. Furthermore, in line with the European Union's priority toward protection and welfare of animals and the ‘Three R’ (Replacement, Reduction, Refinement) initiatives to limit the use of animal models, recent advancements in three-dimensional organoid technology, which mimic or model human tissues, provide a promising alternative to study normal and abnormal developmental in a controlled, human-specific context. Human-specific platforms not only overcome species differences but also pave the way toward precise, personalized insights critical for next-generation stress neurobiology research.

Despite this promise, there is still a finite number of published studies using *in-vitro* human models in the context of stress and psychopharmacology, as highlighted by a recent systematic review (Kirkpatrick et al., 2025). Most of our knowledge of stress and the placenta-brain axis comes from animal models, especially rodents, which has been the subject of many excellent articles (Sze and Brunton, 2024). While human-derived organoid and organotypic culture systems represent a significant advance for modeling human-specific developmental processes, they also have important limitations. Current organoid models often lack the full cellular diversity, vascularization, and immune components present *in vivo*, which are critical for recapitulating the complex interactions of human tissues. Additionally, organoids are limited in their ability to model systemic physiological processes, such as maternal-fetal nutrient exchange, hormonal signaling, and immune interactions, which are essential for understanding pregnancy and neurodevelopment. The important systemic communication aspect of the placenta-brain axis is still missing in these single-organ model systems. For this reason, many research efforts are going into developing organ-on-a-chip and microfluidic systems that can facilitate between-tissues communication (Richards et al., 2024; Mani et al., 2025). Importantly in this context, humanized animal models (for example via genetic manipulation (Yusupov et al., 2024), cell or organoid transplantation (Zhang et al., 2025)) represent new advances used to overcome limitations of human *in vitro* systems. Finally, batch-to-batch variability, limited scalability, and challenges in long-term culture and maturation further constrain their utility. While organoid models are powerful tools for studying human-specific mechanisms especially at the cellular and molecular level, they should be interpreted in the context of these limitations and, importantly, integrated with data from i*n vivo* studies and clinical research.

## 3 Future directions and key questions

Both animal and human studies support that cellular and molecular indicators of mental illness – and resulting changes in endocrine, inflammatory and neurotransmission activity in pregnant women – play key roles in shaping their children's development (Coussons-Read, 2013). Corticosteroids and neurotransmitters are fundamental elements dysregulated in prenatal psychopathology. While lasting effects on child development have been shown (Krontira et al., 2020) through associative evidence, critical questions remain regarding maternal-to-fetal transmission and mechanisms of protection via placenta-specific cells.

To advance our understanding of the placenta-brain axis and especially its involvement in stress transmission and vulnerability, several key questions must be addressed. (1) How does the placenta-brain axis develop in parallel and in interaction to ensure a healthy fetal outcome? (2) How do maternal stressors alter placental function in a sex-specific manner? (3) Can we identify biomarkers in the placenta at birth that predict neurodevelopmental outcomes? The research highlighted herein lays the compelling foundation for framing psychiatric pathology as biological phenomena, where early-life stress and genetic liability intertwine to shape brain development. This framework, integrating neurodevelopment, genetics, and placental biology, provides emerging leaders in the neurobiology of stress with a powerful roadmap to guide transformative future research.

## 4 Conclusion

When it comes to fetal development, the placenta is not merely a support organ that is discarded at birth. It is a dynamic and active participant, including under conditions of maternal perinatal stress. Integrating placental biology into stress and psychopathology research will refine our understanding of disease origins and inform targeted interventions. If we understand that the health of the placenta directly impacts the health of the developing brain in addition to other organs, an interdisciplinary clinical approach could become essential. For example, placental biosamples at birth could serve as biomarkers of future child risk, and treatment of pregnancy or mental disorders in the mother with the developing child in mind could make the distinction between future risk or resilience. As we unravel the complex placenta-brain axis, the path forward lies in embracing human-specific models and mechanistic research that can bridge the gap from discovery to clinical diagnostics or treatment. With these tools and knowledge, one can envision future personalized medicine approaches that utilize model systems to test drug-specific response in a non-invasive manner and even incorporate the fetal or maternal genetic background into the equation. Ultimately, improving our understanding of neuroplacentology will contribute to the shift toward a more developmentally informed approach to stress neuropsychiatry.

## Declaration of competing interest

The authors declare that they have no known competing financial interests or personal relationships that could have appeared to influence the work reported in this paper.

## Data Availability

No data was used for the research described in the article.
